# GeoPep: A Geometry-Aware Masked Language Model for Protein-Peptide Binding Site Prediction

**DOI:** 10.1021/acs.jcim.6c00187

**Published:** 2026-06-29

**Authors:** Dian Chen, Yunkai Chen, Tong Lin, Sijie Chen, Levent Burak Kara, Xiaolin Cheng

**Affiliations:** † Department of Biomedical Engineering, 1466 Johns Hopkins University, Baltimore, Maryland 21218, United States; ‡ College of Pharmacy, 2647 The Ohio State University, Columbus, Ohio 43210, United States; § Department of Machine Learning, School of Computer Science, and Department of Mechanical Engineering, 6612 Carnegie Mellon University, 5000 Forbes Avenue, Pittsburgh, Pennsylvania 15213, United States; ∥ Department of Mechanical Engineering, Carnegie Mellon University, Pittsburgh, Pennsylvania 15213, United States

## Abstract

Multimodal approaches that integrate protein structure and sequence have achieved remarkable success in protein–protein interface prediction. However, extending these methods to protein-peptide interactions remains challenging due to the inherent conformational flexibility of peptides and the limited availability of structural data that hinders direct training of structure-aware models. To address these limitations, we introduce GeoPep, a novel framework for peptide binding site prediction that leverages transfer learning from ESM3, a multimodal protein foundation model. GeoPep fine-tunes ESM3′s rich prelearned representations from protein–protein binding to address the limited availability of protein–peptide binding data. The fine-tuned model is further integrated with a Kolmogorov–Arnold Network (KAN)-based architecture for complex nonlinear approximation. Furthermore, the model is trained using distance-based loss functions that exploit 3D structural information to enhance binding site prediction. Comprehensive evaluations demonstrate that GeoPep significantly outperforms existing methods in protein–peptide binding site prediction by effectively capturing sparse and heterogeneous binding patterns.

## Graphical abstract

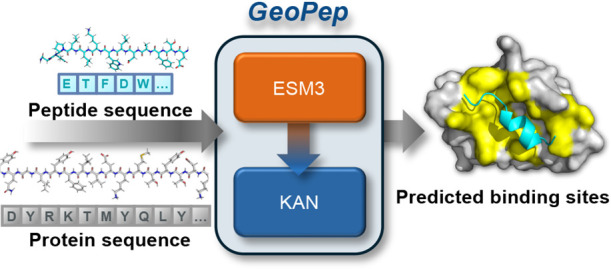

## 1 Introduction

Protein–peptide interactions underpin diverse cellular processes, including signal transduction, immune recognition, and transcriptional regulation, and constitute a major class of therapeutics.^1,2^ Peptides offer a unique pharmacological profile, combining the structural adaptability of small molecules with the specificity of biologics.^3,4^ Unlike small-molecule inhibitors, which require well-defined binding pockets and often exhibit off-target promiscuity, peptides can engage extended protein surfaces through multivalent contacts.^5,6^ At the same time, they circumvent key limitations of biologics, such as poor tissue penetration, immunogenicity, and manufacturing complexity. Compared with biologics, therapeutic peptides show less immunogenicity and have lower production costs,^2^ and they uniquely bridge the gap between small molecules and biologics through their programmable architectures and multifaceted biointerfaces.^3^ These properties render peptides promising candidates for modulating the estimated 80% of disease-associated proteins that remain inaccessible to conventional drug modalities.^4,5^

Despite their therapeutic potential, structural characterization of protein–peptide complexes is challenging.^7,8^ These interactions are frequently transient and involve substantial conformational rearrangements, resulting in scarce high-resolution structural data for protein-peptide complexes.^1,9^ Peptides often undergo disorder-to-order transitions upon binding, as evolution has conferred upon many proteins the capability to partially fold and adopt highly ordered states only in the presence of specific binding partners.^10,11^ Intrinsically disordered proteins are highly flexible and undergo disorder-to-order transitions upon binding, with their flexibility and dynamics combined with extended surface area endowing them with the ability to adapt.^12^ This conformational flexibility, while functionally important, creates additional complexity for computational modeling.^13,14^ The limited availability of experimental data necessitates computational methods to predict peptide binding sites and poses and quantify interaction energetics.^15,16^ Moreover, protein–peptide interaction data sets remain significantly smaller than protein–protein data sets, with many protein targets having fewer than 100 known peptide binders.^17,18^

Traditional physics-based strategies—such as molecular docking and molecular dynamics (MD) simulation—have been widely applied^6,19^ but face fundamental limitations: (1) the inherent conformational flexibility of peptides necessitates exhaustive sampling beyond feasible time scales;^20^ (2) the absence of experimentally determined peptide structures complicates initial pose generation;^6^ (3) inadequate representation of solvation effects introduces systematic errors in binding affinity estimation;^6^ and (4) scoring functions lack discriminative power for native-like conformations versus decoys.^19^ These limitations have motivated the development of machine learning-based approaches.

Deep learning models such as ScanNet and PeSTo have demonstrated strong performance in protein–protein interface prediction.^21,22^ However, they fail to generalize to protein-peptide interactions, which exhibit sparse, localized contact patterns rather than extensive interfacial networks. Recent deep learning methods for protein-peptide interaction prediction have shown promising results,^1,23^ though existing models primarily focus on identifying binding residues on the protein surface and face challenges with class imbalance.^24^ Large-scale protein structure prediction models, including AlphaFold, show variable performance on protein-peptide complexes, with accuracy strongly dependent on the presence of characterized binding motifs. AlphaFold2 achieves 75% accuracy for motif-containing sequences versus 36% accuracy for nonmotif interactions.^7^ The performance of specialized models such as PepNN remains limited due to the paucity of protein-peptide complex structures for training.^25^ Recent advances in protein language models, particularly the ESM3 family of multimodal foundation models, have shown promise, but their application to peptide-specific tasks requires careful adaptation.^26,27^

To address these limitations, we introduce GeoPep, a geometry-aware peptide binding site prediction model that combines transfer learning from protein foundation models with a Kolmogorov–Arnold Network (KAN)-based architecture for flexible nonlinear function approximation. GeoPep fine-tunes ESM3, a multimodal protein foundation model trained on large-scale structural and sequence data,^26^ for peptide binding prediction. ESM3 represents a significant advance as it jointly reasons over protein sequence, structure, and function within a unified framework.^26^ This strategy transfers ESM3′s prelearned protein representations to the peptide domain to overcome the data scarcity limitation in modeling protein-peptide interactions. We train and validate our model using the Propedia database,^18^ a comprehensive repository for protein-peptide identification.

Transfer learning has proven effective in drug discovery contexts, with studies showing up to 8-fold improvements in prediction accuracy while using an order of magnitude less training data.^28–30^ Utilizing AI-driven approaches for drug-target interaction prediction requires large volumes of training data, which are not available for the majority of target proteins,^31^ making transfer learning particularly valuable. Protein language models enable effective transfer learning by adapting foundation models for specific tasks,^28^ and transfer learning enables models to learn common drug-target interaction features from large data sets.^32^

To capture the sparse and heterogeneous interaction patterns characteristic of peptide binding sites, we employ KANs^33^ as expressive nonlinear downstream predictors. KANs have emerged as powerful alternatives to multilayer perceptrons, offering flexible functional representations and improved interpretability.^33–35^ KANs have been successfully applied to bioinformatics tasks, including transcription factor binding site prediction,^36^ and their flexibility and strong modeling capabilities hold great potential in genomics, protein structure prediction, and biological network analysis.^37^ Such expressive nonlinear modeling is particularly valuable when working with limited peptide–protein interaction data.

Furthermore, our framework incorporates distance-based loss functions operating at the structural level to ensure geometric consistency in the binding site prediction. Distance constraints have proven effective in protein structure prediction,^38^ with methods showing that geometric relationships between amino acids can significantly enhance prediction accuracy. Extension of deep-learning-based prediction to interresidue orientations in addition to distances enables the generation of more accurate models.^39^ DeepPotential accurately predicts the distribution of complementary geometric descriptors, including hydrogen-bonding potential,^40^ and state-of-the-art methods utilize distance constraints combined with evolutionary information to achieve unprecedented accuracy.^41^ By enforcing spatial coherence, these constraints ensure that predicted binding sites maintain realistic three-dimensional relationships, addressing a key limitation of sequence-only approaches.^42,43^ Benchmarking across diverse data sets demonstrates that GeoPep achieves state-of-the-art performance, underscoring the utility of foundation model adaptation for peptide-centric prediction tasks.

## 2 Results

### 2.1 GeoPep Architecture for Geometry-Aware Peptide Binding-Site Prediction

GeoPep takes a candidate protein–peptide pair as input and outputs residue-wise binding probabilities for the receptor, representing the confidence that each residue participates in protein–peptide interactions (Figure 1a). The model is formulated as a residue-level interface localization task: rather than determining whether the peptide and protein bind, GeoPep predicts where and how the peptide engages the protein surface. The architecture combines transfer learning from ESM3, a state-of-the-art multimodal protein foundation model trained on billions of sequences and structures to jointly reason over protein sequence, structure, and function, with sequential Kolmogorov–Arnold Network (KAN) modules. During training, GeoPep further incorporates distance-based constraints to enforce spatial coherence in binding predictions (Figure 1b).

**Figure 1:**
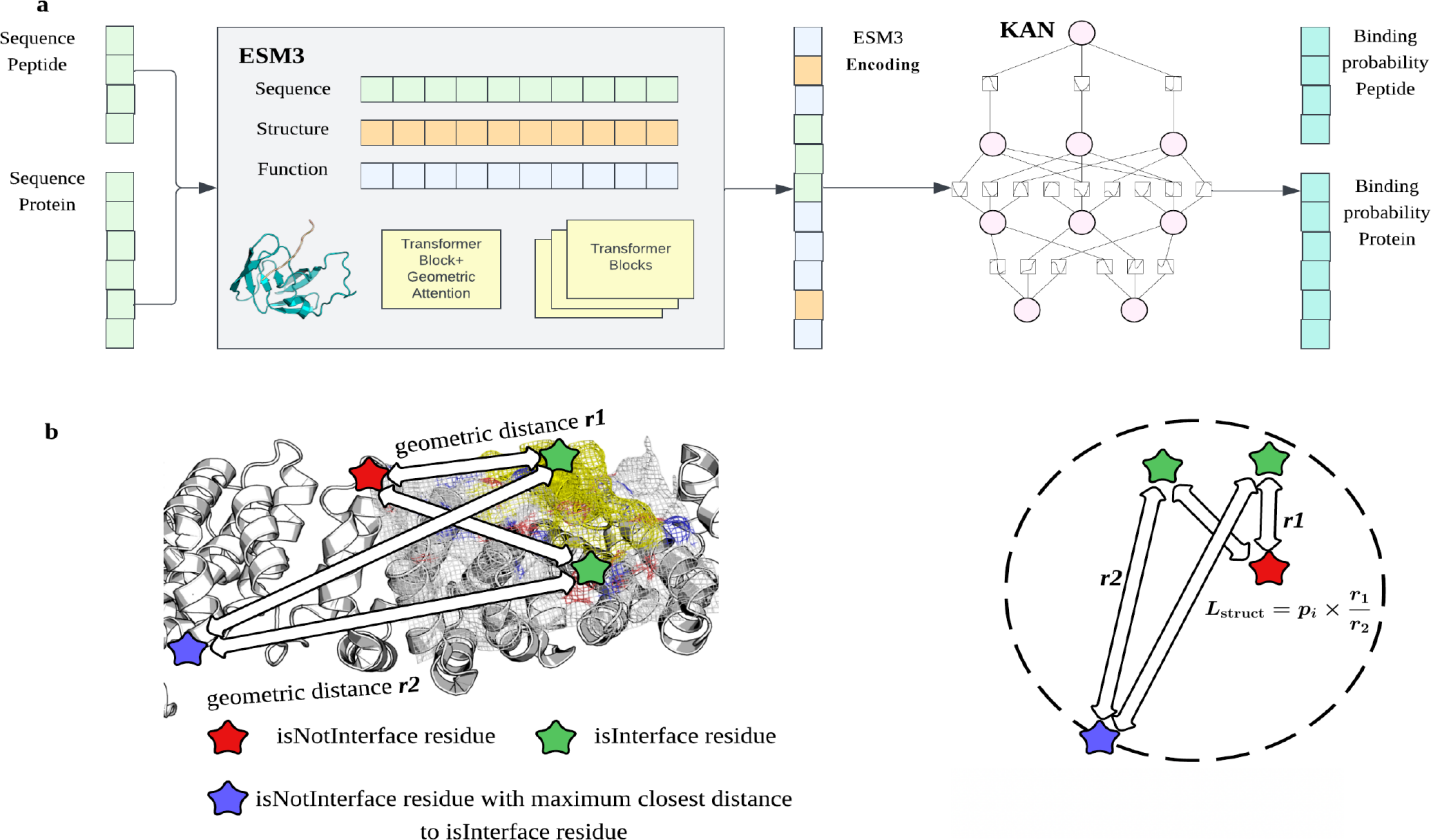
(a) GeoPep architecture combining ESM3 transfer learning with Kolmogorov–Arnold Network (KAN) modules for peptide binding site prediction. Peptide and protein sequences are processed through ESM3′s multimodal encoder, which integrates sequence, structural, and functional information via transformer blocks with geometric attention mechanisms. The resulting ESM3 embeddings are passed to KAN layers that employ learnable B-spline activation functions to model complex nonlinear binding patterns, outputting residue-level binding probabilities. (b) Distance-based geometric loss function enforcing spatial consistency in binding site predictions. For each noninterface residue *i* incorrectly predicted as a binding site (false positive), *r*_1_ = *d*_3D_(*i*) represents the minimum 3D distance to the nearest true interface residue, and *r*_2_ = max­(*d*_3D_) represents the maximum such distance across all false positives in the complex. The geometric loss is computed as *p*_*i*_ × (*r*_1_/*r*_2_), where *p*_*i*_ denotes the predicted binding probability for residue *i*. This distance-normalized penalty ensures that high-confidence false positives farther from true binding sites incur larger penalties, guiding the model toward spatially coherent predictions clustered around verified interface residues. Detailed mathematical formulations are provided in Methods.

In GeoPep, ESM3 embeddings, which capture evolutionary and structural knowledge through geometric attention mechanisms modeling three-dimensional spatial relationships among amino acids, are fed into sequential KAN modules. Unlike conventional multilayer perceptrons with fixed activation functions, KANs employ learnable activation functions on network edges, providing a more flexible functional representation for modeling complex nonlinear relationships. This design allows the model to capture intricate peptide–protein interaction patterns while maintaining a lightweight adaptation layer when fine-tuning large foundation models. Such a design is particularly suitable for peptide binding prediction, where training data are relatively limited and expressive nonlinear modeling is required.

Distance-based loss functions are implemented to enforce spatial coherence of the predicted binding surface. These geometric constraints penalize configurations where residues assigned high binding confidence are geometrically distant from the peptide. This constraint improves localization fidelity and reduces reliance on large labeled data sets by injecting physically meaningful structures into the learning objective. The resulting predictions form contiguous, spatially plausible binding patches on the receptor surface, consistent with peptide–protein interaction topologies.

### 2.2 Comparative Analysis of ESM2 and ESM3 Foundation Models in GeoPep

ESM2 and ESM3 represent successive generations of protein foundation models with distinct capabilities. ESM2 is a sequence-only transformer trained via masked language modeling and lacks structural context. In contrast, we use the open-source ESM3_sm_open_v0 variant, a 1.4-billion-parameter model that integrates sequence, structure, and functional information, enabling richer representations for the peptide binding prediction.

To quantify the impact of structural signals on peptide binding prediction, we implemented GeoPep with both ESM2 and ESM3 as backbone models, maintaining identical downstream components, including KAN modules and distance-based loss functions. Both variants were fine-tuned on the same training data sets under identical hyperparameter configurations to ensure a fair comparison. Model performance was evaluated on a test data set of 4436 protein-peptide complexes collected from PPIRef after filtering, spanning diverse binding poses with experimentally validated interface annotations.^44^

The comparative evaluation demonstrates the performance advantage of ESM3-GeoPep across all of the metrics (Figure 2). In the ROC analysis (Figure 2a, left), ESM3-GeoPep achieves an AUC of 0.945 compared to 0.889 for ESM2-GeoPep, indicating improved discriminative ability for distinguishing binding from nonbinding residues. The precision-recall curves (Figure 2a, right) reveal a more pronounced difference, with ESM3-GeoPep attaining an AUC of 0.748 versus that of 0.639 for ESM2-GeoPep.This improvement in the high-precision regime is particularly relevant for binding site identification under class imbalance, where minimizing false positives is critical. Analysis of the 3D distance-based loss (Figure 2b) shows that ESM3-GeoPep produces predictions with a substantially lower mean distance loss of 0.039 compared to ESM2-GeoPep at 0.361, with reduced variance in the distribution. This indicates that multimodal structural representations encoded in ESM3 improve the spatial localization of predicted binding residues relative to the peptide. Visualization of binding site predictions on representative complexes from the test data set (Figure 2c) illustrates these quantitative differences: ESM3-GeoPep (bottom row) exhibits more concentrated true positive predictions (blue) and decreased false positive predictions (red) around the peptide (yellow) compared to ESM2-GeoPep (top row).

**Figure 2:**
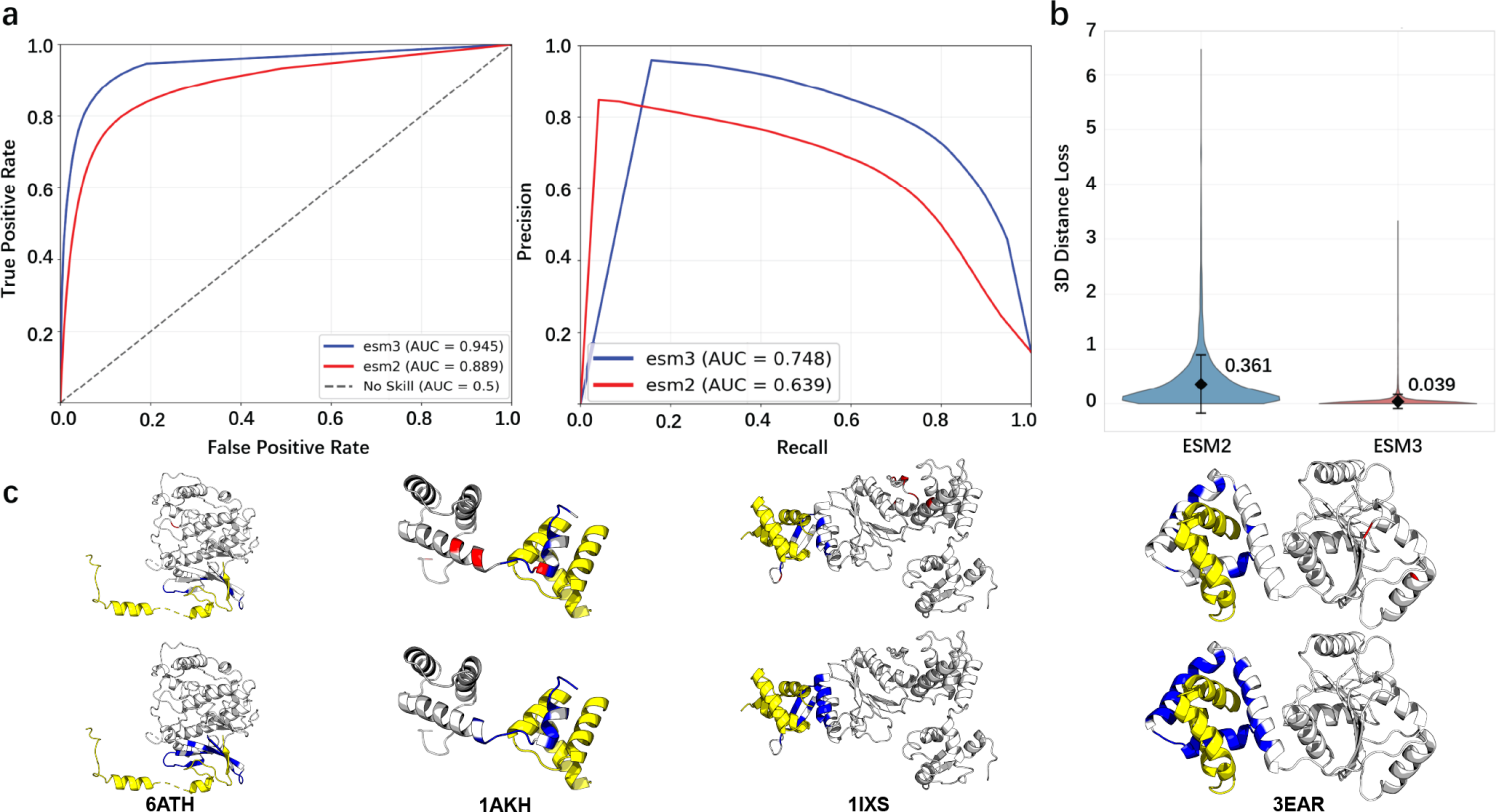
(a) ROC and precision-recall curves comparing GeoPep performance using ESM2 versus ESM3 backbones. (b) Distributions of 3D distance-based loss for ESM2 and ESM3, with the calculation methodology detailed in Methods. (c) Binding site prediction visualizations for ESM2 (top) and ESM3 (bottom) on representative peptide–protein complexes (PDB IDs: 6ATH, 1AKH, 1IXS, and 3EAR).^45–48^ Peptides are colored yellow, and true positive residues are highlighted in blue and false positives in red.

### 2.3 Kolmogorov–Arnold Networks Provide Expressive Functional Modeling

To evaluate the architectural contribution of KAN layers, we compared GeoPep variants using KAN modules versus traditional MLP modules on the test dataset while keeping all other settings identical (Figure 3a–c). The precision-recall analysis (Figure 3a) shows that KAN-based GeoPep achieves an AUC of 0.748 compared to 0.725 for the MLP variant, indicating improved discriminative ability, particularly in the high-precision regime. Analysis of the 3D distance-based loss (Figure 3b) reveals that KAN produces predictions with substantially lower mean distance loss (0.039) compared to MLP (0.875), with a narrower distribution indicating more consistent spatial localization of predicted binding residues. Visualization on PDB 8UCS from test data set (Figure 3c) illustrates these quantitative differences: the KAN variant (top) exhibits more true positive predictions (blue) around the peptide (yellow) with zero false positives (red) compared to the MLP variant (bottom). These results demonstrate that KAN’s learnable activation functions provide advantages over fixed-activation MLPs in both classification performance and geometric accuracy for peptide binding site prediction.

**Figure 3:**
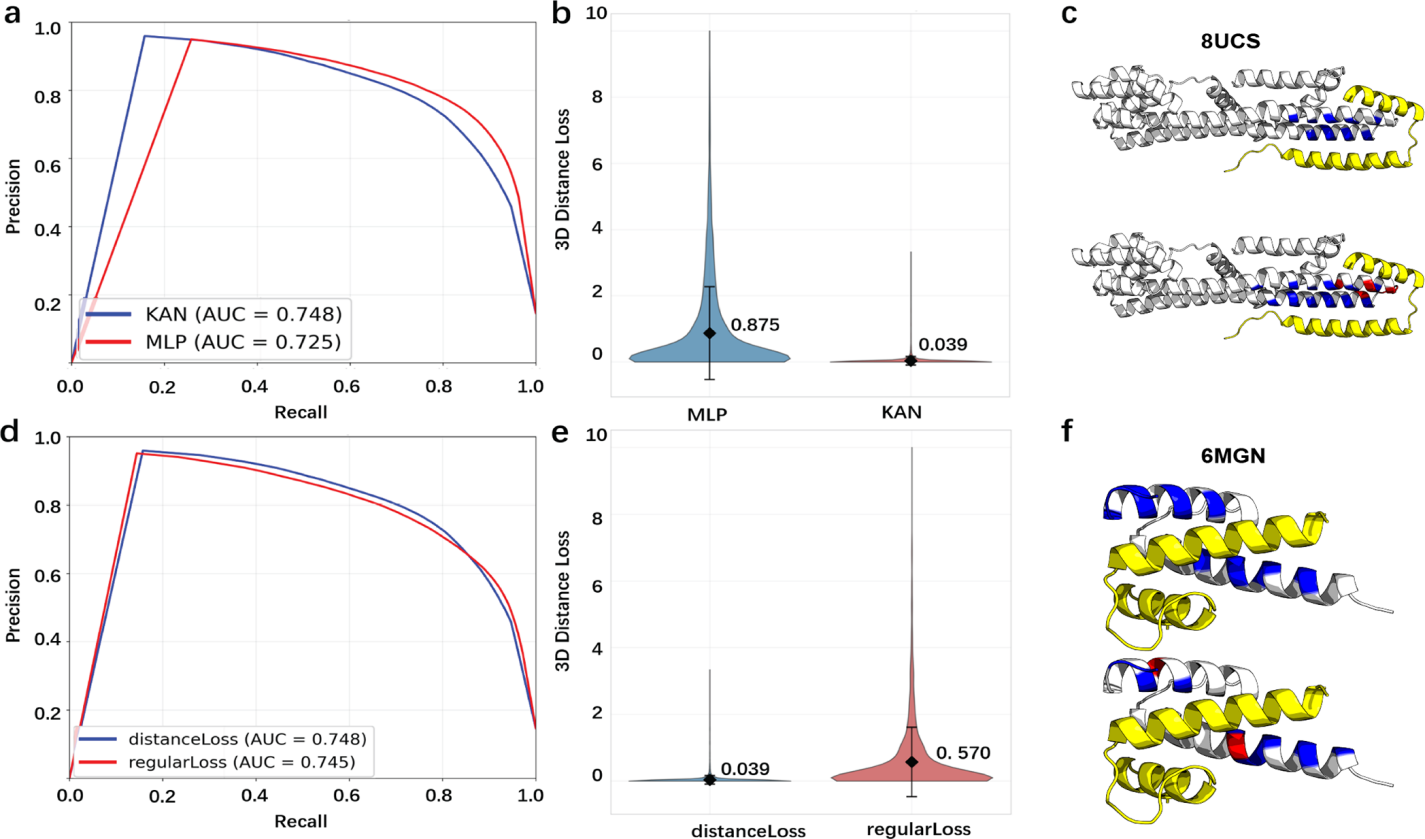
(a) Precision-recall curves comparing KAN versus MLP architectures in GeoPep, with KAN achieving AUC of 0.748 versus 0.725 for MLP. (b) Distributions of 3D distance-based loss for architectural comparisons, showing KAN (mean = 0.039) outperforming MLP (mean = 0.875), with calculation methodology detailed in Methods. (c) Representative binding site prediction visualizations for PDB 8UCS,^49^ showing predictions from KAN (top) and MLP (bottom). (d) Precision-recall curves comparing distance-based versus standard loss functions in GeoPep, with distanceLoss achieving AUC of 0.748 versus 0.745 for regularLoss. (e) Distributions of 3D distance-based loss for loss function comparisons, showing distanceLoss (mean = 0.039) outperforming regularLoss (mean = 0.570), with calculation methodology detailed in Methods. (f) Representative binding site prediction visualizations for PDB 6MGN,^50^ comparing distance-based loss (top) and standard loss (bottom) configurations.

### 2.4 Distance-Based Geometric Constraints Improve Spatial Coherence

In addition to the standard cross-entropy loss for residue-level classification, GeoPep incorporates distance-based geometric constraints to enforce spatial coherence in the predicted binding sites. While cross-entropy loss minimizes prediction errors for individual residues, it does not capture the spatial relationships among the binding residues. The geometric loss penalizes configurations where high-confidence predictions are distant from the peptide, encouraging the model to learn spatially coherent binding patterns rather than isolated-residue classifications.

To evaluate its impact, we compared GeoPep variants trained with and without geometric regularization on test data sets. The precision-recall analysis (Figure 3d) shows comparable classification performance, with the distance-based loss variant achieving an AUC of 0.748 versus 0.745 for the standard loss configuration. Analysis of the 3D distance-based loss (Figure 3e) reveals that the distance-based loss variant produces predictions with substantially lower mean distance loss (0.039) compared to the standard loss configuration (0.570), with a markedly narrower distribution, indicating more consistent spatial localization. Visualization on PDB 6MGN from the test data set (Figure 3f) shows that the distance-based loss configuration (top) produces spatially concentrated predictions closer to the peptide, while the standard loss configuration (bottom) exhibits more dispersed false positive predictions. These results suggest that while the two configurations achieve comparable classification performance as measured by PR-AUC, the distance-based geometric constraint substantially improves the spatial coherence and proximity of the predicted binding residues to the true interface.

### 2.5 Component Ablation of GeoPep

To quantify the contribution of each design component, we performed ablation experiments on the external PPIRef test set (Figure 4). The full model achieved the best protein-side AUPRC of 0.748, indicating that the combination of multimodal pretraining, expressive prediction heads, and geometric regularization is beneficial for protein–peptide interface prediction.

**Figure 4:**
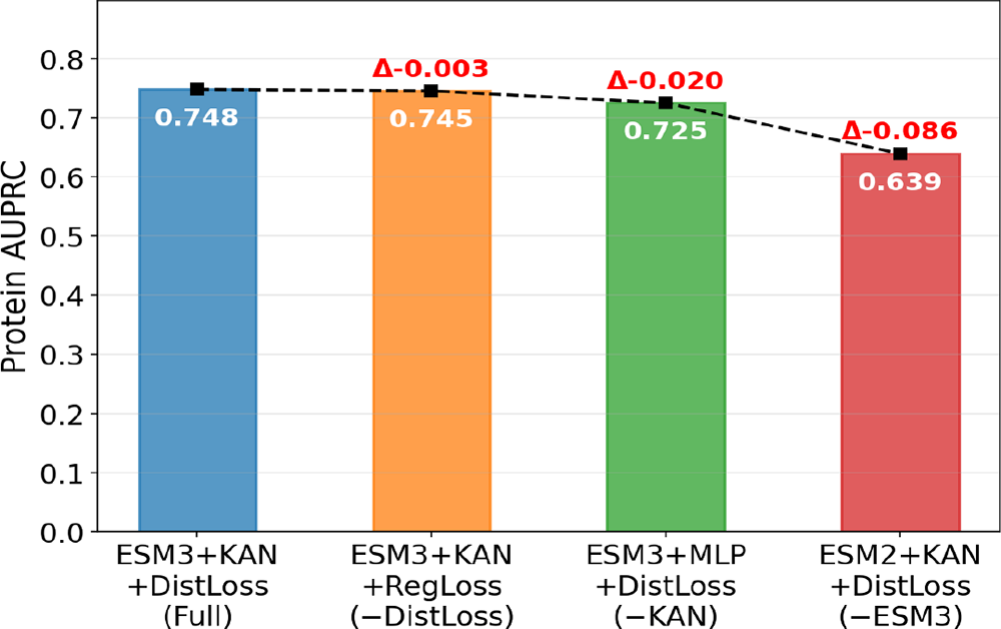
Component ablation of GeoPep on the external PPIRef test set.

Replacing ESM3 with ESM2 caused the largest reduction (0.639), highlighting the importance of the multimodal pretraining. This result suggests that structural information learned during pretraining substantially improves the residue-level recognition of peptide-binding patterns beyond sequence-only representations.

Replacing the KAN prediction head with a conventional MLP also reduced performance (0.725), indicating that flexible nonlinear parametrization is advantageous for modeling the complex determinants of peptide recognition. Although both heads operate on the same pretrained embeddings, the KAN architecture appears to extract more informative decision boundaries from transferred representations.

Removing geometric regularization produced a smaller but consistent decrease (0.745), supporting the complementary role of the weak structural supervision. While the effect on AUPRC is modest, distance-aware training improved spatial coherence, resulting in more localized interface predictions.

Overall, these results indicate that GeoPep benefits from complementary gains at the representation, prediction head, and supervision levels.

### 2.6 GeoPep Outperforms Existing Binding-Site Prediction Methods

We benchmarked GeoPep against leading binding site prediction methods, including PepNN,^25^ ScanNet,^21^ and PeSTo,^22^ using the PPIRef test data set (Figure 5a–d).

**Figure 5:**
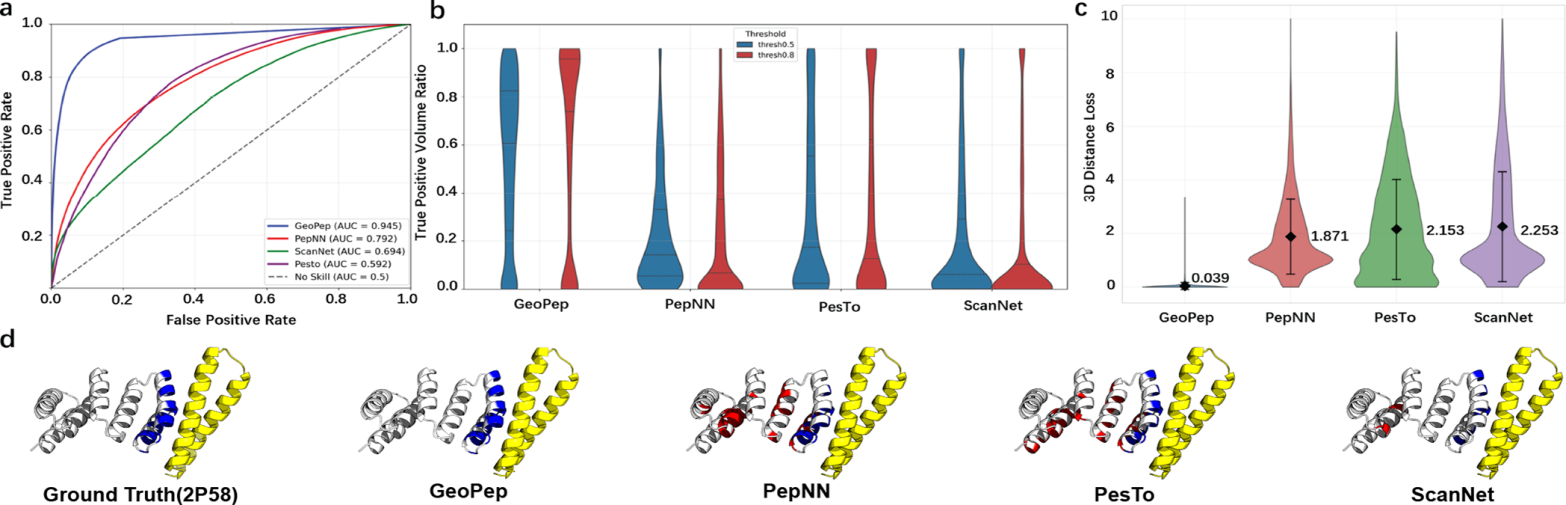
(a) ROC curves comparing GeoPep with baseline methods (PepNN, PesTo, ScanNet). (b) True positive volume ratio (TPVR) analysis at two thresholds (0.5 and 0.8), calculated as the ratio of true positive volume to total predicted volume; higher ratios indicate more accurate and contiguous binding surface predictions. (c) Distributions of 3D distance-based loss across methods, with calculation methodology detailed in Methods. Lower values correspond to better geometric accuracy in binding site predictions. (d) Visualization of peptide–protein interface predictions for the 2P58.^51^ Ground truth interface residues are shown in the leftmost panel. For predictions by GeoPep, PepNN, Pesto, and ScanNet, peptides are colored yellow, true positives are highlighted in blue, and false positives in red.

Unless otherwise specified, residues with predicted probability *p*_*i*_ ≥ 0.8 were classified as binding sites, whereas residues with *p*_*i*_ < 0.8 were considered nonbinding. This threshold follows commonly used benchmark settings in prior binding-site prediction studies, where probability outputs are typically interpreted as confidence scores and analyses focus on high-confidence predictions rather than model-specific optimal cutoffs.^22,25^ The same threshold was applied uniformly across all methods to ensure fair comparison. For completeness, we additionally report model performance under a more permissive threshold (*p*_*i*_ ≥ 0.5). The true positive volume ratio (TPVR) analysis further demonstrates that GeoPep maintains high spatial coherence of predicted binding surfaces across different probability thresholds (Figure 5b). Overall, the threshold of 0.8 provides a practical trade-off between prediction confidence and coverage in protein–peptide interface prediction, a sparse task in which true binding residues constitute only a small fraction of the protein surface.

The ROC analysis (Figure 5a) demonstrates that GeoPep achieves an AUC of 0.945, substantially outperforming PepNN (0.792), ScanNet (0.694), and PeSTo (0.592). ScanNet and PeSTo, both designed for protein–protein binding site prediction, show limited discriminative capability for protein–peptide prediction tasks. PepNN, designed specifically for protein–peptide complexes, performs better than the protein–protein methods but remains below GeoPep. The true positive volume ratio (TPVR) analysis (Figure 5b), which measures the degree to which true positive residues densely fill their minimal enclosing surface volume on the protein, reveals that GeoPep maintains high ratios at both 0.5 and 0.8 probability thresholds, indicating more continuous and spatially coherent binding surface predictions compared to baseline methods that show greater variability. Analysis of the 3D distance-based loss (Figure 5c) confirms GeoPep’s geometric advantage: GeoPep achieves the lowest mean distance loss (0.039) with a narrow distribution, compared to PepNN (1.871), PeSTo (2.153), and ScanNet (2.253), all of which exhibit higher values and greater variance. Visualization of predictions on PDB 2P58 (Figure 5d) illustrates these quantitative differences: GeoPep produces predictions closely matching the ground truth interface (leftmost panel), with concentrated true positives (blue) around the peptide (yellow) and zero false positives (red), whereas PepNN, PeSTo, and ScanNet show more dispersed predictions with increased false positive rates.

Table 1 further highlights the advantage of GeoPep, demonstrating consistently superior performance across all four evaluation metrics. GeoPep achieves the highest precision (0.8379), F1 score (0.7174), and accuracy (0.9279) among all compared methods.

**Table 1:** Performance Comparison of Peptide Binding Site Prediction Methods at Probability Threshold = 0.8

**model**	**precision**	**recall**	**F1-score**	**accuracy**
GeoPep	0.8379	0.6272	0.7174	0.9279
PepNN	0.5056	0.3479	0.4122	0.8552
PeSTo	0.4562	0.5634	0.5042	0.7644
ScanNet	0.4376	0.3386	0.3818	0.7713

Collectively, these results demonstrate that combining multimodal transfer learning from ESM3 with KAN-based nonlinear functional modeling and geometry-aware loss functions enables robust modeling of peptide–protein interactions.

### 2.7 Structural Evaluation

To assess the performance of GeoPep in localizing protein–peptide interfaces without explicit structural input, we examined three representative complexes from the test set, ordered by increasing interface-residue ratio, defined as

$$\mathrm{Interface}\;\mathrm{Ratio}=\frac{N_{\mathrm{interface}}}{N_{\mathrm{total}}}$$ 1

where *N*_interface_ is the number of receptor residues annotated as interface residues, and *N*_total_ is the total number of receptor residues. As shown in Figure 6a, *Left (6WLW)* corresponds to the human V-ATPase complex, in which approximately 6.7% of receptor residues are annotated as interface residues.^52^ A short helical peptide engages an elongated groove-like surface on the receptor, and GeoPep correctly identified the complete interface without false positive predictions. *Middle (7Z31)* depicts the yeast RNA polymerase III–Ty1 integrase complex, where approximately 11.7% of receptor residues form the annotated interface.^53^ In this asymmetric and topologically complex setting, GeoPep recovered the complete contact patch without false positives or false negatives. *Right (7WZ6)* presents the MyoD–E47 heterodimer, in which approximately 43.4% of receptor residues are annotated as interface residues and two helices associate through a broader coiled-coil-like interface.^54^ For this larger and more distributed interaction surface, GeoPep correctly captured the expanded contact region with complete agreement with the annotated interface. Together, these examples suggest that GeoPep generalizes across protein–protein complexes spanning low-, medium-, and high-interface-residue regimes, while maintaining accurate residue-level localization across groove-mediated, surface-exposed, and helix–helix recognition modes.

**Figure 6:**
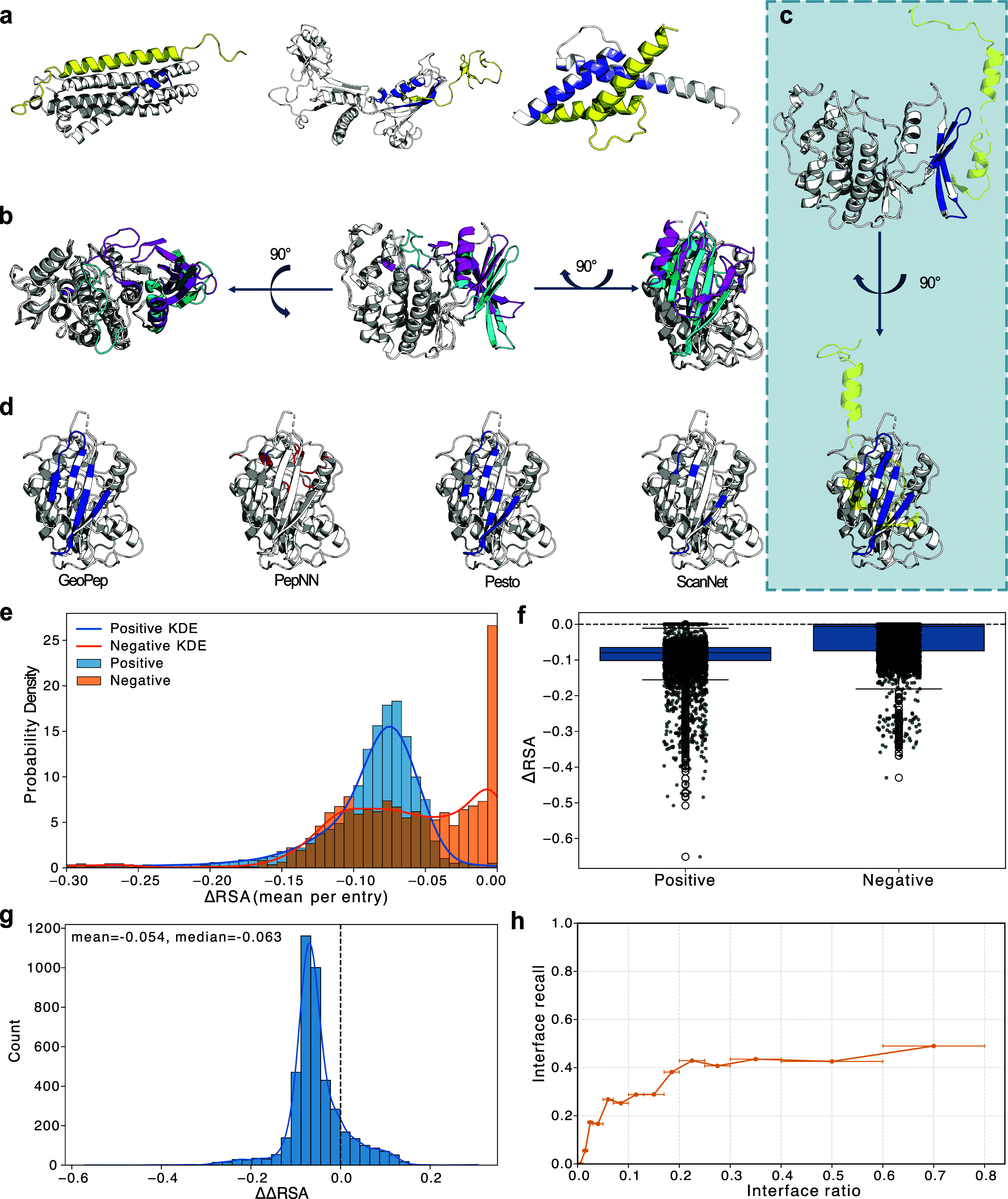
(a) Case studies of peptide–protein interface prediction with 100% residue-level accuracy; peptides are shown in yellow and correctly predicted interface residues in blue. Left to right: 6WLW, 7Z31, 7WZ6 (interface-residue count increases from low to moderate to high). (b) Overlay of the apo (4EK3, magenta) and holo (6ATH, cyan) states of HIV protease illustrating conformational change upon peptide binding, shown from three viewpoints (center, −90° horizontal rotation, and +90° vertical rotation) for structural clarity. (c) Holo state rotated by 90° about the indicated axis for structural clarity. (d) GeoPep predictions on 6ATH alongside three baselines (PepNN, Pesto, and ScanNet). (e) Per-class normalized histograms and kernel density estimates (KDEs) for Positive (blue) and Negative (orange) predictions, highlighting ΔRSA distributions. (f) Distributions of per-entry ΔRSA for Positive vs Negative predictions. (g) Distribution of per-entry differences between the mean ΔRSA of residues predicted as Positive and those predicted as Negative. (h) Protein-level interface recall plotted against interface ratio.

We next evaluated GeoPep’s performance in scenarios where peptide binding is coupled to substantial protein conformational changes. As shown in Figure 6b, for the apo–holo states of CDK2 (PDBs: 4EK3 for apo; 6ATH for the peptide-bound complex),^55,56^ extensive structural rearrangements accompany binding of cyclin A and the p27-derived peptide. Across the three orthogonal views, the dominant conformational differences can be grouped into three mobile regions: (i) a helical segment spanning residues 46–57; (ii) a lateral β-sheet region formed by residues 17–37 and 66–81; and (iii) a flexible loop comprising residues 152–171. Together, these coordinated displacements indicate that partner recognition in CDK2 involves broader backbone reorganization across multiple structural elements rather than a purely local pocket-adjustment event. Figure 6c shows the ground-truth interface of the holo complex from two orthogonal views for structural reference. The experimentally annotated interface is concentrated along the exposed β-sheet surface that engages cyclin A and the p27-derived peptide, providing a spatial template for interpreting the prediction results shown in Figure 6d. Despite the absence of the bound-state conformation in the apo structure, the structure-less GeoPep model correctly identifies the interaction-prone region as interfacial, demonstrating that the model is not restricted to rigid, preorganized sites but can accommodate binding-coupled structural rearrangements that often challenge geometry-based scoring functions.

In this induced-fit case, GeoPep was compared with established baseline methods, including PepNN, PeSTo, and ScanNet (Figure 6d). GeoPep concentrates predictions on the experimentally annotated interface and recovers the dominant β-sheet binding surface with the strongest overall performance (precision = 1.000, recall = 0.969; 63 true positives and 0 false positives). By comparison, PeSTo and ScanNet also achieve perfect precision but with substantially lower recall (0.876 and 0.692, respectively), indicating incomplete recovery of the annotated interface, whereas PepNN yields lower precision (0.722) together with lower recall (0.600), reflecting both missed interface residues and off-target predictions.

To test whether GeoPep predictions align with underlying biophysical principles, we analyzed residue-level changes in relative solvent accessibility (RSA), defined as

$${\mathrm{RSA}}_{i}=\frac{{\mathrm{SASA}}_{i}}{{\mathrm{SASA}}_{i}^{\max}}$$ 2

where SASA_*i*_ is the solvent-accessible surface area of residue *i*, and SASA_*i*_^max^ is the maximal accessible area for that residue type. RSA is dimensionless and ranges from 0 to 1. We then quantified binding-induced changes as

$$\Delta \mathrm{RSA}={\mathrm{RSA}}_{\mathrm{holo}}-{\mathrm{RSA}}_{\mathrm{apo}}$$ 3

A defining feature of true interface residues is burial in the bound state. As shown in Figure 6e, residues predicted as Positive by GeoPep are shifted toward more negative ΔRSA values, whereas predicted Negatives cluster closer to zero, indicating minimal changes in solvent exposure.

At the complex level (Figure 6f), per-entry mean ΔRSA values show consistent separation across 4344 complexes, with predicted Positives exhibiting stronger burial (mean = −0.0915, median = −0.0798) than predicted Negatives (mean = −0.0373, median = −0.0042). For each complex *k*, we further quantified the within-complex separation as

$$\Delta \Delta {\mathrm{RSA}}^{\left(k\right)}={\overset{®}{\Delta \mathrm{RSA}}}_{\mathrm{Positive}}^{\left(k\right)}-{\overset{®}{\Delta \mathrm{RSA}}}_{\mathrm{Negative}}^{\left(k\right)}$$ 4

where ${\overset{®}{\Delta \mathrm{RSA}}}_{\mathrm{Positive}}^{\left(k\right)}$ and ${\overset{®}{\Delta \mathrm{RSA}}}_{\mathrm{Negative}}^{\left(k\right)}$ denote the mean ΔRSA values of residues predicted as Positive and Negative, respectively. The resulting per-complex ΔΔRSA values form a unimodal distribution centered below zero (mean = −0.0543, median = −0.0632; Figure 6g). A one-sided Wilcoxon signed-rank test (Pratt method) confirmed the significance of this shift (*W* = 1, 070, 382, *p* < 10^–300^), with a large effect size (*r* = 0.77). Bootstrap 95% confidence intervals excluded zero (mean difference [−0.0561, −0.0524]; median [−0.0644, −0.0622]).

Together, these results indicate that GeoPep preferentially assigns positive labels to residues that undergo binding-associated burial, consistent with a core biophysical hallmark of protein–peptide recognition.

As shown in Figure 6h, protein-side interface recall increases with the interface ratio. Small or sparse interfaces yield lower recall, likely reflecting weaker interaction signals and stronger class imbalance. In contrast, larger and more contiguous interfaces are recovered more reliably with recall approaching a plateau for medium-to-high interface ratio complexes. This trend suggests that the model preferentially captures coherent interaction patches with stronger learnable patterns.

For the induced-fit case (PDB: 6ATH), GeoPep’s strong performance is consistent with the statistical trends in Figure 6e–g and the interface-size dependence shown in Figure 6. The experimentally annotated interface combines substantial binding-associated burial, a contiguous multiresidue contact surface, and a relatively large interface extent, all of which favor accurate recovery by the model. By not presupposing a preformed pocket, GeoPep remains effective on induced-fit and binding-coupled refolding systems, where conformational rearrangements often reduce the accuracy of purely structure- or geometry-driven predictors.

## 3 Discussion

Our results demonstrate that GeoPep effectively leverages multimodal protein foundation models for protein–peptide binding-site prediction through a combination of transfer learning, expressive downstream predictors, and geometric regularization. The superior performance of ESM3 over ESM2 suggests that multimodal pretraining with structural information provides clear advantages for capturing the geometric constraints underlying peptide recognition. This finding highlights the value of incorporating structure-aware representations even when downstream prediction is performed from sequence-derived embeddings.

The integration of distance-based geometric regularization further improves the spatial coherence of the predicted binding residues. Compared with purely classification-based objectives, the additional geometric constraint encourages predictions that are more consistent with realistic three-dimensional residue arrangements, thereby reducing spatially implausible positives and improving the interface localization. More broadly, this result suggests that weak structural supervision can enhance residue-level interaction prediction without requiring explicit docking or a complex generation during inference.

We further find that Kolmogorov–Arnold Networks (KANs) provide an effective prediction head for fine-tuning large protein foundation models. In our experiments, KAN-based architectures achieve comparable or improved performance relative to conventional MLP-based alternatives, indicating that flexible functional parametrizations can be advantageous for modeling nonlinear determinants of peptide binding.

A notable implication of these findings is that effective peptide interface prediction does not necessarily require explicit complex generation during inference. Instead, pretrained biomolecular representations combined with lightweight task-specific adaptation can already recover substantial binding information at the residue resolution.

The amino acid composition of predicted receptor-side interface residues is also consistent with the known biophysical preferences of peptide-binding surfaces. Aromatic and interaction-favoring residues such as Tyr, Trp, and Met are enriched, whereas less favorable residues such as Pro are depleted (Figure S1). This agreement further supports the biological plausibility and interpretability of the GeoPep predictions.

Interestingly, exploratory random negative-sampling did not improve model performance (Figure S2). One possible explanation is that GeoPep is optimized for residue-level prediction, where native complexes already provide abundant noninterface residues as negative supervision. Because randomly mismatched peptide–receptor pairs do not accurately reflect realistic nonbinding interactions and show limited physicochemical divergence from native complexes, their inclusion may introduce label noise and reduce training effectiveness (Figure S3).

To quantify residual homology at the chain level, we computed for each PPIRef complex the maximum peptide identity and maximum receptor identity to the Propedia training set, as well as the joint identity. The resulting peptide-only, receptor-only, and joint identity distributions (Figure S4A) indicate that no retained complex shows a joint receptor–peptide sequence identity ≥ 50*%* to any Propedia training complex, consistent with the benchmark’s design goal of avoiding trivially redundant complexes while still reflecting realistic levels of single-chain homology encountered in external evaluations. To further assess robustness under stricter redundancy criteria, we constructed multiple nonredundant subsets by applying reduced identity thresholds separately to the receptor chain, the peptide chain, or the joint identity. We then recalculated AUROC on each subset (Figure S4B): receptor-only <50*%* (AUROC = 0.873), peptide-only <50% (AUROC = 0.807), joint identity <30% (AUROC = 0.820), receptor-only <30% (AUROC = 0.796), and peptide-only <30% (AUROC = 0.748). As expected, progressively stricter thresholds yield smaller—and more challenging—evaluation sets; nevertheless, GeoPep maintains competitive predictive performance across all tested redundancy definitions, supporting its generalization beyond trivial near-duplicate complexes. Notably, performance degradation is more pronounced under peptide-only filtering than under receptor-only filtering. For example, the peptide-only <50% subset yields performance comparable to the much stricter receptor-only <30% subset. This trend suggests that peptide sequence novelty imposes a stronger generalization challenge than receptor sequence novelty, likely reflecting the short length and motif-driven nature of peptide-mediated recognition, where relatively modest peptide divergence can substantially alter local interaction patterns and binding determinants.

Taken together, GeoPep consistently outperforms both specialized protein–peptide predictors and general protein–protein interface methods across multiple benchmarks. These results highlight the broader potential of combining large-scale pretrained biomolecular representations with lightweight task-specific adaptation for molecular interaction modeling.

Beyond binary binding-site prediction, the GeoPep framework may be extended to related tasks such as residue–residue contact prediction, protein–peptide complex modeling, and binding-affinity estimation. Integration with the peptide design or therapeutic discovery pipelines may further enable the rational optimization of peptide binders, inhibitors, and modulators.

## 4 Limitations

Despite these promising results, several limitations should be acknowledged. First, currently available protein–peptide structural data sets remain substantially smaller than general protein–protein interaction resources, which may limit generalization across rare binding modes, receptor families, or underrepresented interface topologies. Continued data set expansion and improved redundancy control will be important for future progress.

Second, the current training data consists primarily of naturally occurring linear peptides. Consequently, GeoPep does not explicitly model structural constraints associated with cyclic peptides, disulfide-bonded peptides, stapled peptides, or chemically modified residues. Extending the prediction to conformationally constrained or noncanonical peptide classes remains an important direction for future work.

Third, the construction of informative negative examples remains an open challenge for protein–peptide learning. While exploratory random negative pairing did not improve performance in our experiments, more realistic hard negatives may be required for robust training.

In particular, useful hard negatives may involve structurally compatible surfaces, homologous receptors, alternative binding pockets, docking-derived non-native poses, or peptides with similar physicochemical properties but incorrect binding specificity. Incorporating experimentally validated nonbinding protein–peptide pairs would be especially valuable for robust benchmarking and future model development.

Addressing these limitations will further improve the model robustness, biological realism, and applicability in therapeutic peptide discovery.

## 5 Method

### 5.1 Data Set

ESM3 was pretrained on a large-scale multimodal data set comprising protein sequences and structures. The sequence data included UniRef (2023_02 release, 156 M sequences at 90% clustering), MGnify90 (2023_02 release, 621 M sequences), JGI (2029 M sequences at 70% clustering), and OAS (1192 M antibody sequences at 95% clustering). The structural data incorporated PDB structures (203 K chains prior to May 1, 2020), AlphaFoldDB v4 (69 M structures at 90% clustering), and ESMAtlas (v0 and v2023_02, 179 M structures at 90% clustering).

For fine-tuning ESM3 on protein–peptide binding prediction, we constructed a data set from Propedia v2.3, which aggregates protein–peptide complexes from the Protein Data Bank. Complexes were filtered to include peptides of length 2–50 residues and proteins resolved by X-ray crystallography at resolution <2.5 Å or by NMR spectroscopy. Binding residues were defined as residues within 6 Å of any peptide atom. Additional filtering retained complexes with peptides longer than 10 residues and protein chains shorter than 500 residues, yielding 32,290 protein–peptide chain combinations from 12,540 unique complexes. The native protein–peptide complexes were randomly split into training (90%, 29,061 instances) and validation (10%, 3229 instances) subsets.

For independent evaluation, we additionally constructed an external test set from PPIRef, a curated structural resource of protein–protein interaction complexes derived from the Protein Data Bank.^44^ Following the peptide definition used throughout this study, peptide chains were operationally defined as chains containing 2–50 residues. Candidate entries were filtered to retain peptide chains of length 2–50 residues and receptor chains with the additional constraint that receptor length was greater than or equal to peptide length. To reduce redundancy with the Propedia training data, candidate receptor–peptide pairs were compared against the Propedia training set using MMseqs2 easy-search. A candidate complex was removed if both the receptor chain and peptide chain matched a training example with sequence identity ≥50%. The remaining nonredundant complexes were used as an external test set for all primary benchmark evaluations. In total, 4436 complexes satisfied these criteria and were retained for external evaluation.

To further assess train-test redundancy, we examined the distributions of peptide identity, receptor identity, and joint identity relative to the Propedia training set (Figure S4A). Sequence identities were computed independently using MMseqs2 easy-search. Joint identity was defined as the minimum of the peptide-chain and receptor-chain identity values for each complex. No retained complex exhibited joint receptor–peptide identity ≥ 50*%* relative to any training example. We additionally evaluated GeoPep on stricter nonredundant subsets generated using receptor-only, peptide-only, and joint sequence identity thresholds of <50% or <30% (Figure S4B).

In separate exploratory experiments, we also evaluated a simple random pairing strategy for constructing synthetic negative peptide–receptor pairs, but this strategy was not adopted in the final training pipeline. Additional details are provided in the Supporting Information.

### 5.2 Input Preparation

Input structures were processed using BioPython to extract atomic coordinates and sequence information from PDB files. Peptide sequences were padded to 50 residues and protein sequences to 500 residues to ensure consistent batch dimensions. Protein–peptide sequence pairs were concatenated into a single sequence using a chain-break separator token (|) between the peptide and protein, and then tokenized using ESM3's sequence tokenizer. The | token is used to indicate two interacting chains, allowing the model to capture cross-chain relationships via self-attention.

### 5.3 Model Architecture

Input peptide–protein sequence pairs are processed through the ESM3_sm_open_v0 model (1.4-billion parameters), a multimodal protein foundation model that encodes molecular representations via multihead self-attention mechanisms across sequence, structural, and functional modalities.

These residue-level embeddings, each of dimension 1536, are passed to a Kolmogorov–Arnold Network (KAN) prediction module to compute binding probabilities for individual protein residues. Each KAN layer implements learnable activation functions parametrized as B-spline basis functions with trainable coefficients, enabling flexible modeling of complex nonlinear relationships. The final layer applies a softmax activation to produce per-residue binding confidence scores, normalized to the range [0, 1], representing the predicted probability of each residue belonging to the binding interface. GeoPep is formulated as a residue-level interface localization task for a given protein–peptide pair. The objective is to predict where and how the peptide engages the receptor surface, rather than to determine whether the two molecules bind.

For discrete binding-site prediction, residues with predicted probability ≥0.8 are classified as binding residues, while residues with predicted probability <0.8 are considered nonbinding. Predictions are obtained directly by thresholding the residue-level probabilities, without additional spatial clustering or postprocessing.

### 5.4 Training Objective

The composite loss combines binary cross-entropy for residue-level classification with a distance-based geometric regularization term:

$$L_{\mathrm{total}}=L_{\mathrm{CE}}+\lambda L_{\mathrm{struct}}$$ 5

The cross-entropy loss for binary classification is

$$L_{\mathrm{CE}}=-\frac{1}{N}\overset{N}{\underset{i=1}{\sum }}\left[y_{i}\log\left(p_{i}\right)+\left(1-y_{i}\right)\log\left(1-p_{i}\right)\right]$$ 6

where *y*_*i*_ ∈ {0, 1} indicates whether residue *i* is a true binding residue, *p*_*i*_ is the predicted probability of residue *i* being a binding residue, and *N* is the total number of residues.

The structural regularization term penalizes false positives proportionally to both confidence and distance from true binding sites:

$$L_{\mathrm{struct}}=\frac{1}{N}\overset{N}{\underset{i=1}{\sum }}L_{\mathrm{struct}}\left(i\right)$$ 7

$$L_{\mathrm{struct}}(i)=\{\begin{array}{ll} 0 & \mathrm{if}\;y_{i}=1\\ p_{i}\times \frac{d_{3D}(i)}{\max(d_{3D})} & \mathrm{if}\;y_{i}=0 \end{array}$$ 8

where *d*_3D_(*i*) = min_*j*∈*I*_interface__∥**r**_*i*_ – **r**_*j*_∥_2_ is the minimum 3D distance from residue *i* to any true binding residue, *I*_interface_ denotes the set of true binding residues, max­(*d*_3D_) = max_*i*=1_^*N*^ *d*_3D_(*i*) is the maximum 3D distance across all residues. λ is a hyperparameter controlling the regularization strength. Unless otherwise specified, we empirically set λ = 10.0 throughout all experiments. This regularization term enforces spatial coherence by discouraging high-confidence predictions far from true interfaces.

### 5.5 True Positive Volume Ratio

To evaluate binding surface continuity, we introduce the true positive volume ratio metric that quantifies the spatial coherence of predicted binding sites. The convex hull algorithm constructs the smallest convex polytope enclosing all input points, capturing the spatial extent of residue clusters. Let *R*_TP_ denote true positive residues, and *R*_pred_ denote all predicted binding residues. The convex hull volumes are

$$V_{\mathrm{TP}}=\mathrm{ConvexHull}\left(\left\{r_{i}:i\in R_{\mathrm{TP}}\right\}\right)$$ 9

$$V_{\mathrm{pred}}=\mathrm{ConvexHull}\left(\left\{r_{i}:i\in R_{\mathrm{pred}}\right\}\right)$$ 10

where **r**_*i*_ represents the mass center of residue *i*, *V*_TP_ is the volume enclosed by true positive residues, and *V*_pred_ is the volume enclosed by all predicted residues. The true positive volume ratio is

$$\mathrm{TP}\mathrm{Volume}\mathrm{Ratio}=\frac{V_{\mathrm{TP}}}{V_{\mathrm{pred}}}$$ 11

Since *R*_TP_ ⊆ *R*_pred_, this ratio ranges from 0 to 1. Higher ratios indicate compact, continuous binding surfaces where false positives cluster near true positives. Lower ratios suggest spatially dispersed predictions with fragmented binding sites.

### 5.6 Training

The model is trained to predict the peptide binding sites on protein surfaces. The GeoPep architecture was trained on 8 NVIDIA RTX A5000 GPUs using PyTorch Lightning with an FSDP (fully sharded data parallel) distributed training strategy. The training objective employs a composite loss function combining binary cross-entropy loss for residue-level binding classification and the distance-based regularization loss. Gradient descent with automatic mixed precision was used to optimize the 1.4 billion model parameters.

### 5.7 Evaluation

Our method was compared with PepNN, ScanNet, and PeSTo to assess performance on protein-peptide binding site prediction. PepNN is a specialized deep learning method designed for protein-peptide interface prediction, serving as the most relevant baseline for our task. ScanNet and PeSTo are state-of-the-art geometry-based deep learning methods developed for protein–protein interface prediction, including evaluating the transferability of protein–protein models to peptide binding tasks. The benchmarking of GeoPep was performed using an independent external test set constructed from PPIRef, comprising nonredundant protein–peptide instances collected from unique structural complexes after sequence-identity filtering against the Propedia training set.

## Supplementary Material



## Data Availability

The implementation of GeoPep, including the model architecture and training pipeline, is publicly available at https://github.com/Dian0212/GeoPep. Pretrained model weights will be made available upon publication.

## Notes

Amino acid enrichment analyses of predicted receptor interface residues; evaluation of random negative sampling strategies during model training; physicochemical complementarity analyses of native and decoy peptide–receptor pairs; and sequence redundancy analyses and additional external benchmark evaluations under multiple peptide-, receptor-, and joint-identity filtering criteria (PDF)

## ⊥

Amazon, Seattle, Washington 98109, United States
