# Supplementary material for: GeoPep: A Geometry-Aware Masked Language Model for Protein-Peptide Binding Site Prediction
Source: J Chem Inf Model. 2026 Jun 29;66(13):7377–89. doi: 10.1021/acs.jcim.6c00187 (PMC13370878; doi:10.1021/acs.jcim.6c00187)
Supplement: Supplementary file 1 [file ci6c00187_si_001.pdf]

Supporting Information

for

GeoPep: A geometry-aware masked language model for protein-peptide binding site prediction

Dian Chen,[1] Yunkai Chen,[2] Tong Lin,[3,*] Sijie Chen,[2]
Levent Burak Kara,[4] and Xiaolin Cheng[2]

[1] Department of Biomedical Engineering, Johns Hopkins University, Baltimore, MD 21218, USA
[2] College of Pharmacy, The Ohio State University, Columbus, OH 43210, USA
[3] Amazon, Seattle, WA 98109, USA; *This work is unrelated to work at Amazon.
[4] Department of Mechanical Engineering, Carnegie Mellon University, Pittsburgh, PA 15213, USA

Corresponding author: Prof. Xiaolin Cheng, cheng.1302@osu.edu

A

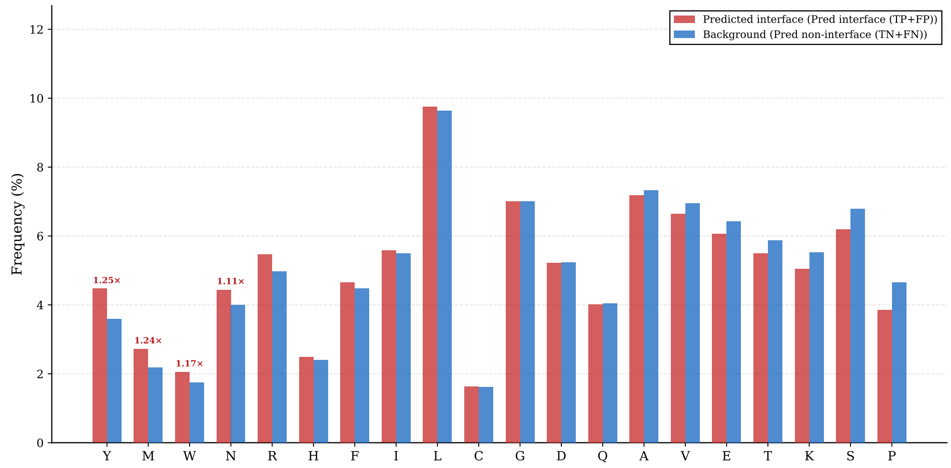

B

	Y	M	W	N	R	H	F	I	L	C	G	D	Q	A	V	E	T	K	S	P
**Interface (%)**	4.47	2.73	2.06	4.44	5.46	2.49	4.65	5.58	9.76	1.63	7.01	5.22	4.01	7.19	6.65	6.06	5.49	5.04	6.19	3.86
**Background (%)**	3.59	2.19	1.76	3.99	4.98	2.40	4.48	5.50	9.63	1.62	7.02	5.24	4.05	7.33	6.95	6.43	5.88	5.53	6.79	4.66
**Enrichment**	**1.25×**	**1.24×**	**1.17×**	**1.11×**	1.10×	1.04×	1.04×	1.01×	1.01×	1.00×	1.00×	1.00×	0.99×	0.98×	0.96×	0.94×	0.93×	0.91×	0.91×	0.83×

Figure S1: Amino acid enrichment analysis of predicted receptor interface residues. **(A)** Frequency distribution of the 20 standard amino acids among predicted interface residues (red) versus background non-interface residues (blue) on the receptor side, sorted by enrichment ratio in descending order. Enrichment values $\geq 1.10\times$ are annotated above each pair of bars. **(B)** Corresponding enrichment table showing the percentage frequency of each amino acid in the predicted interface and background groups, with enrichment ratios highlighted for residues enriched $\geq 1.10\times$. Predicted interface residues are defined as positions with model prediction = 1 (TP+FP). Aromatic residues Tyr (1.25×) and Trp (1.17×) show the strongest enrichment, consistent with their known roles in hydrophobic packing and π–π interactions at peptide–protein interfaces. Met (1.24×) and Asn (1.11×) are also enriched, reflecting contributions from hydrophobic contacts and hydrogen bonding, respectively. Conversely, Pro (0.83×) and Ser (0.91×) are depleted, consistent with their limited capacity for stable interface interactions.

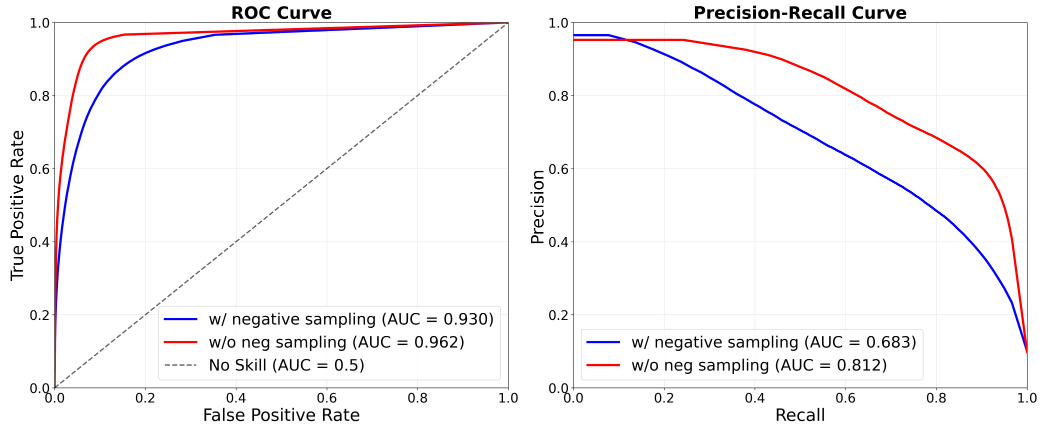

Figure S2: Effect of random negative sampling on GeoPep performance. Random negative sampling refers to the generation of 5,000 synthetic peptide–receptor pairs by randomly mismatching peptides and receptors from different complexes; these synthetic negatives were used only during training and were not included in validation or test evaluation. Comparison of models trained with and without exploratory random negative sampling on the Propedia validation split is shown. **(Left)** Receiver operating characteristic (ROC) curves for protein-side residue prediction. Removing random negative sampling improves AUROC from 0.930 to 0.962. **(Right)** Precision–recall (PR) curves for the same models. Removing random negative sampling improves AUPRC from 0.683 to 0.812. These results indicate that naive sequence-level random negatives do not benefit residue-level interface learning and may introduce noisy supervision relative to training on native complexes alone.

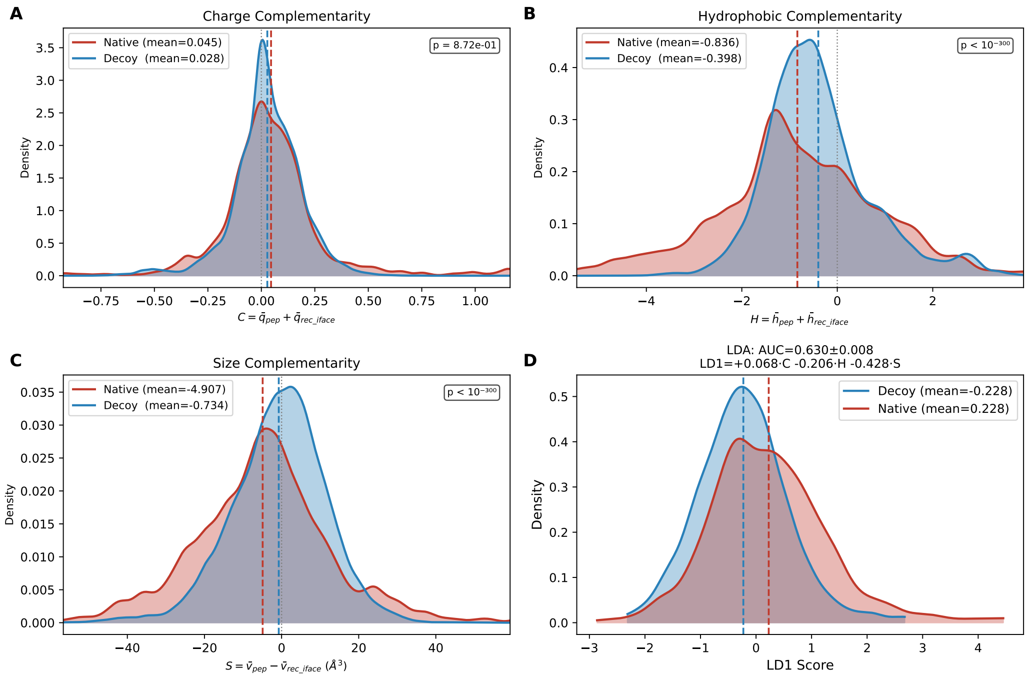

Figure S3: Physicochemical complementarity distributions for native and decoy peptide–receptor interface pairs. For each pair, three signed complementarity metrics are computed: $C = \bar{q}_{\text{pep}} + \bar{q}_{\text{rec\_iface}}$ (charge), $H = \bar{h}_{\text{pep}} + \bar{h}_{\text{rec\_iface}}$ (hydrophobicity), and $S = \bar{v}_{\text{pep}} - \bar{v}_{\text{rec\_iface}}$ (Å^3) (size). **(A–C)** Kernel density estimates of C, H, and S for native (red) and decoy (blue) pairs. Dashed vertical lines indicate group means; p-values are from two-sided Mann–Whitney U tests. Hydrophobicity and size show clearer distributional shifts, whereas charge exhibits substantial overlap. **(D)** Linear Discriminant Analysis (LDA) combining all three features. The LD1 score distributions for native and decoy pairs are shown. The modest separation (5-fold cross-validated AUC = 0.630 ± 0.008) suggests that randomly paired decoys capture only partial physicochemical differences relative to native binding pairs.

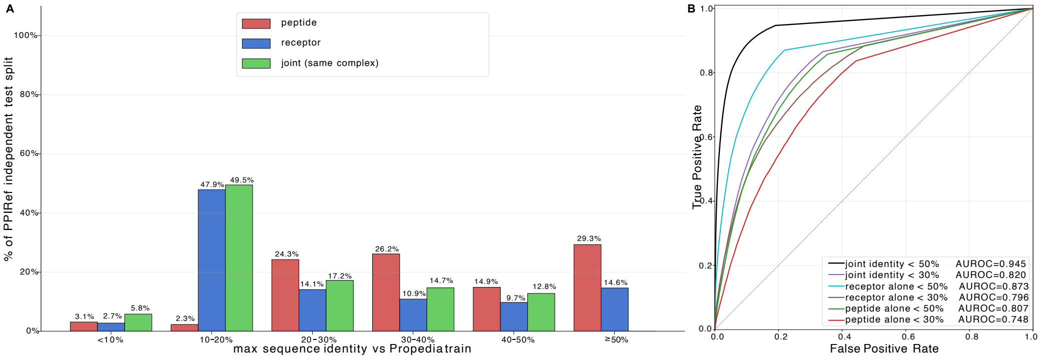

Figure S4: **(A)** Distribution of maximum sequence identity between retained PPIRef external test complexes and the Propedia training set. Red bars indicate peptide-chain comparisons, blue bars indicate receptor-chain comparisons, and green bars indicate joint complex-level comparisons, where peptide and receptor chains originate from the same retained complex. Sequence identities were computed independently using MMseqs2 easy-search. Joint identity was defined as the minimum of the peptide-chain and receptor-chain identity values for each complex. **(B)** ROC curves of GeoPep evaluated on the original PPIRef external benchmark and additional nonredundant subsets generated using peptide-only, receptor-only, and joint sequence identity thresholds relative to the Propedia training set. The original benchmark (black, AUROC = 0.945) corresponds to the primary external test split used throughout this study. Additional stricter subsets include "joint identity $< 30\%$" (purple, AUROC = 0.820), "receptor alone $< 50\%$" (cyan, AUROC = 0.873), "receptor alone $< 30\%$" (brown, AUROC = 0.796), "peptide alone $< 50\%$" (green, AUROC = 0.807), and "peptide alone $< 30\%$" (red, AUROC = 0.748).
